# Supplementary material for: Identification of MYOM2 as a candidate gene in hypertrophic cardiomyopathy and Tetralogy of Fallot, and its functional evaluation in the Drosophila heart
Source: Dis Model Mech. 2020 Dec 18;13(12):dmm045377. doi: 10.1242/dmm.045377 (PMC7758640; doi:10.1242/dmm.045377)
Supplement: Supplementary information [file dmm-13-045377-s1.pdf]

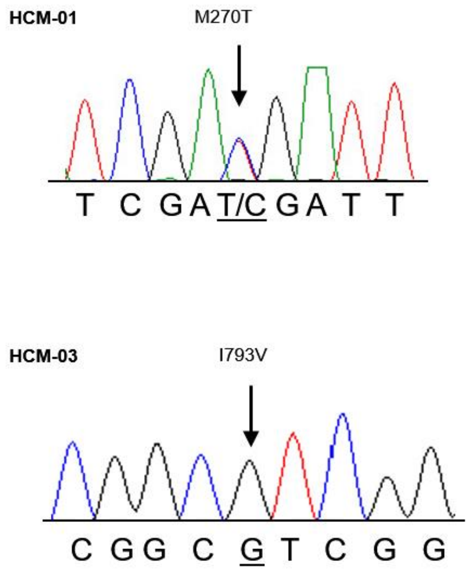

Fig. S1: *MYOM2* mutations in patients with hypertrophic cardiomyopathy (HCM). Sequence electropherograms representing the four mutations in the *MYOM2* gene. The panel shows the mutated sequence demonstrating heterozygosity in three of them (HCM-01, HCM-02, HCM-04). In patient HCM-03, a homozygous mutation was found. The arrow points to the mutated nucleotide.

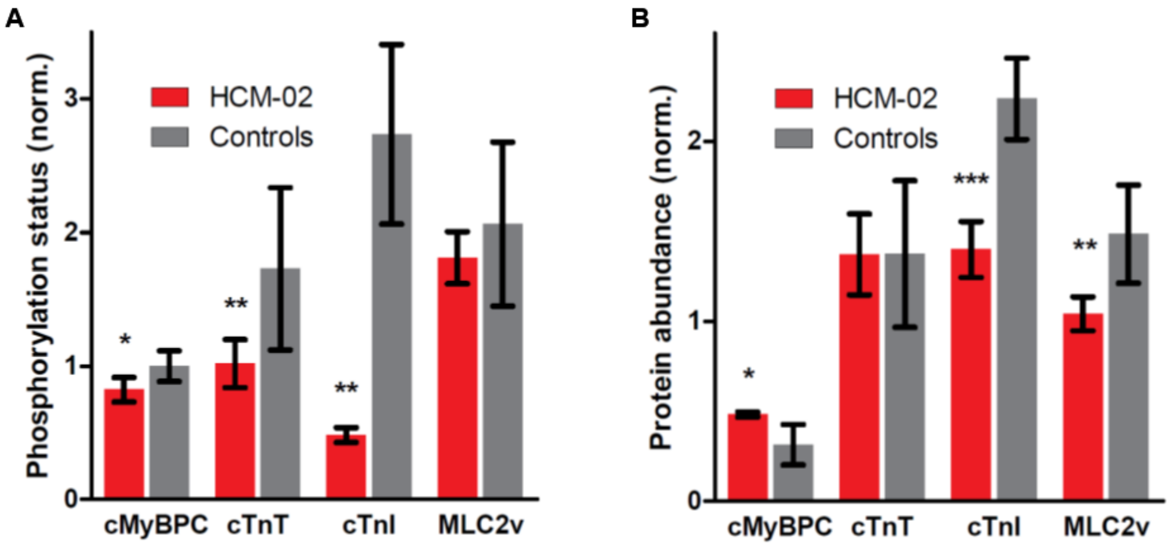

Fig. S2: Analysis of phosphorylation status and protein levels of native myocardial tissue from HCM-02 and controls. (A) Protein phosphorylation was analyzed by calculating the ratio of Pro-Q Diamond staining (phosphorylated protein) vs. SYPRO Ruby staining (total amount of protein; Fig. 2B) for each band, respectively, as described in Kraft et al., J Mol Cell Cardiol 57 (2013). (B) For analysis of relative protein quantities, the bands of the respective proteins on the SYPRO Ruby stained gels (Fig. 2B) were densitometrically analyzed and normalized to the α-actinin signal in the same lane. n=5 different controls; n=5 independent analyses for HCM-02; error bars indicate SD; *p<0.05; **p<0.01, ***p<0.001 (Student's t-test).

Disease Models & Mechanisms • Supplementary information

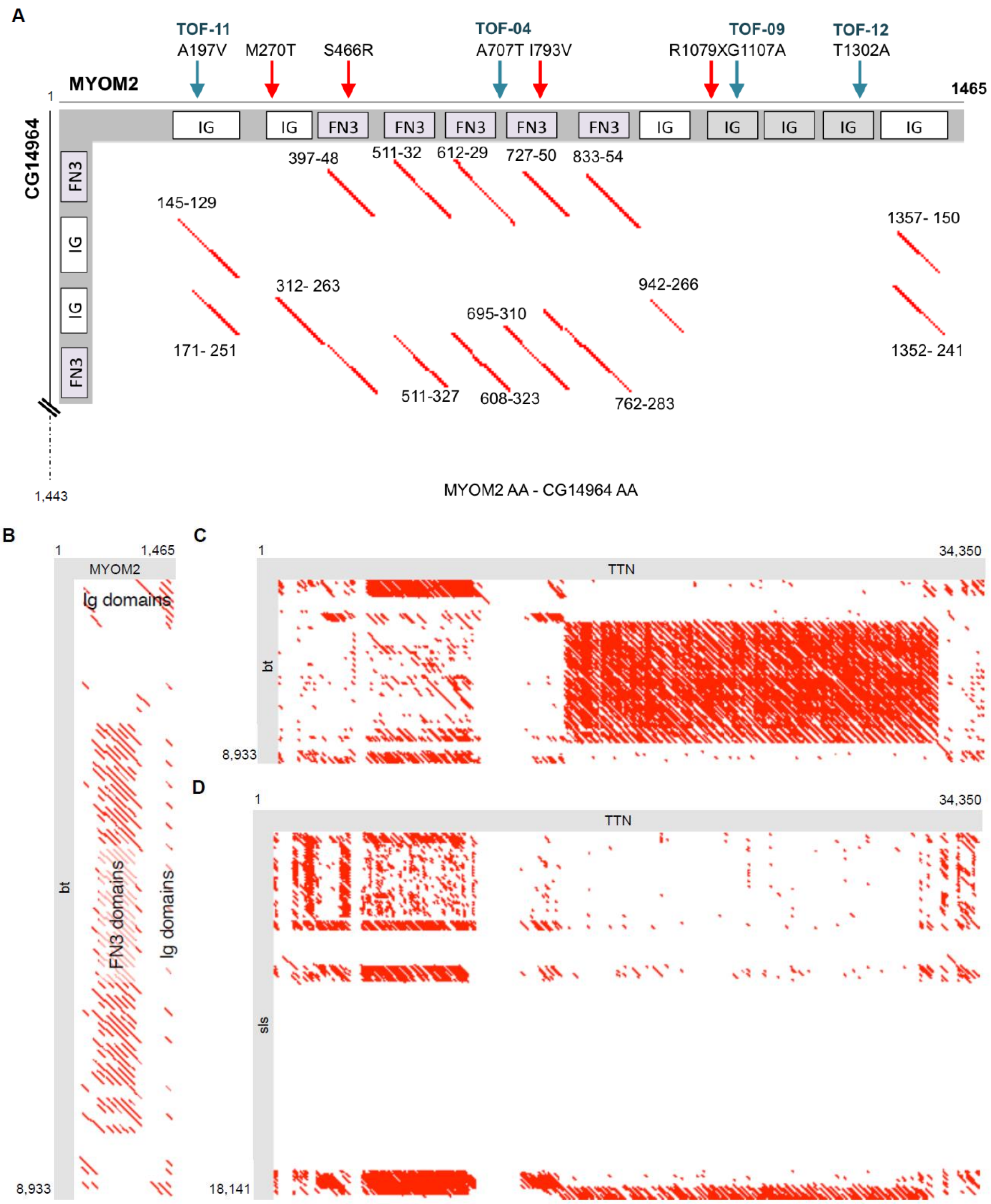

Fig. S3: Amino acid alignments of *Drosophila* orthologs CG14964, bt and sls and human MYOM2 and TTN. (A) Alignment of CG14964 and MYOM2 show that both proteins are composed of fibronectin type III-like (FN3) and immunoglobulin-like (Ig) domains. Arrows indicate mutations in TOF (blue) and HCM patients (red), respectively. (B) Alignment of bent (bt) and MYOM2 comprises a different structure organization with many FN3 domains for bt spanning most of the protein while MYOM2 contains only five FN3 domains in the center. (C) Alignment of bt with TTN shows the conservation of intercalation of FN3 and Ig domains. (D) Alignment of sallimus (sls) also shows the conserved intercalation of FN3 and Ig domains. Alignments indicated in red mainly consist of FN3 and Ig-like domains.

Disease Models & Mechanisms • Supplementary information

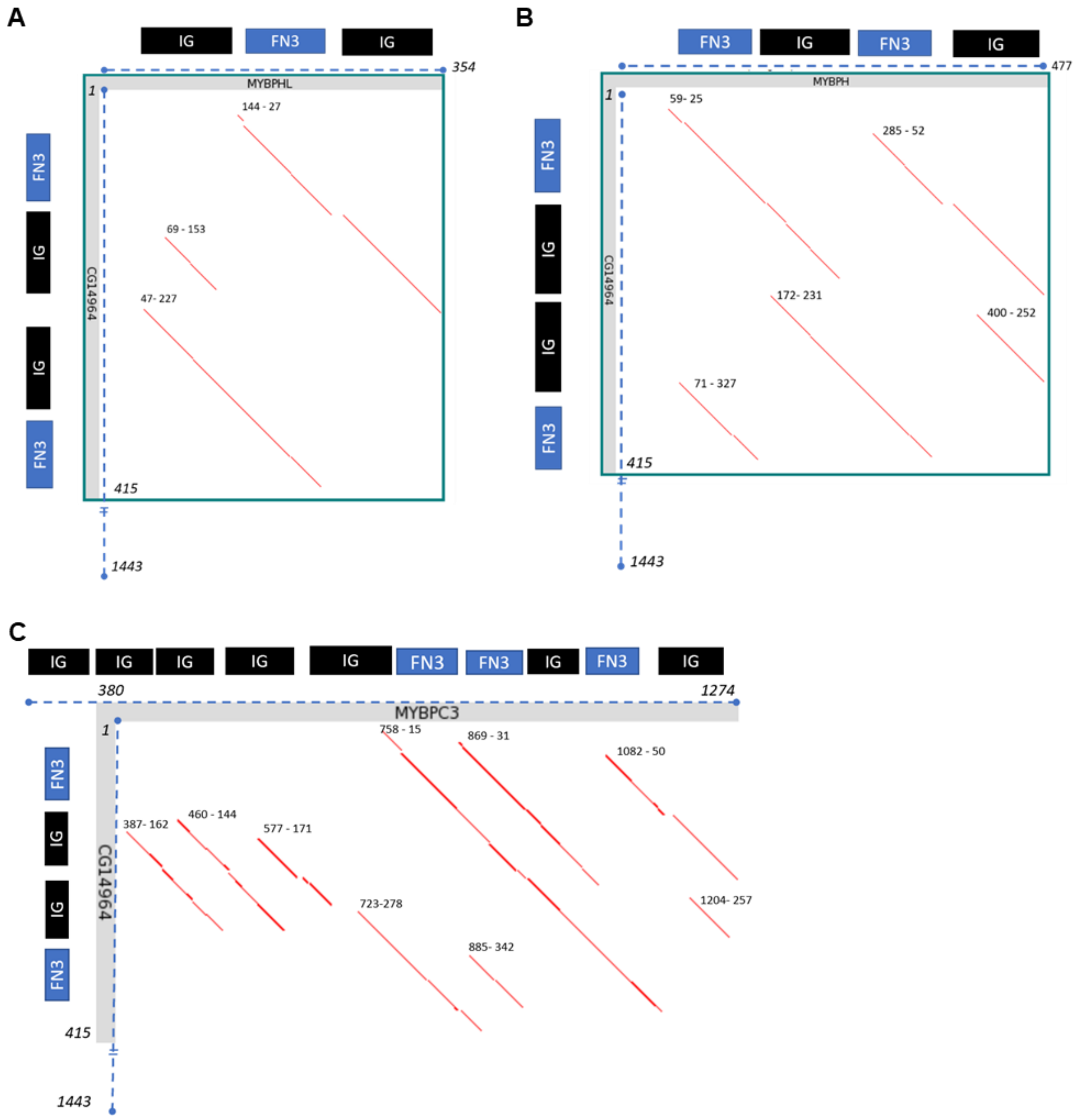

Fig. S4: Protein alignments of CG14964 with (A) MYPHL, (B) MYBPC3 and (C) MYBPH. Alignments show structural similarities between fly and human proteins.

Disease Models & Mechanisms • Supplementary information

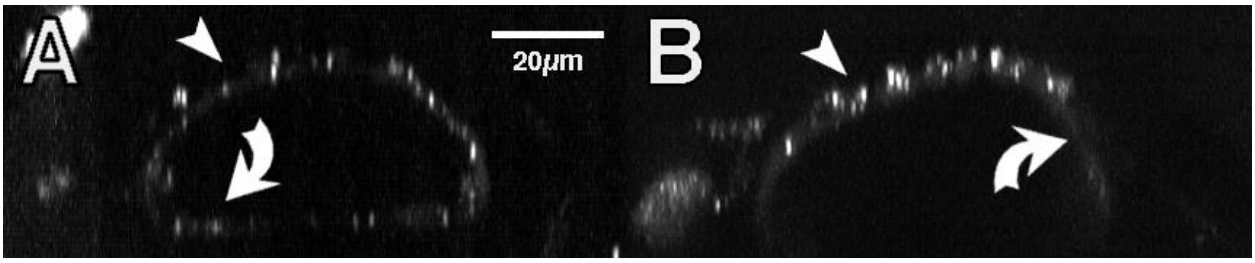

Fig. S5: RNAscope *in situ* for CG14964 in (A) Hand4.2 > GD control and (B) Hand4.2 > CG14964^{HMC06031} RNAi. Note that expression is detectable in ventral layer muscle syncytium (arrowheads) but absent in knockdown cardiomyocytes (curved arrow in B) compared to controls (curved arrow in A), indicating probe specificity.

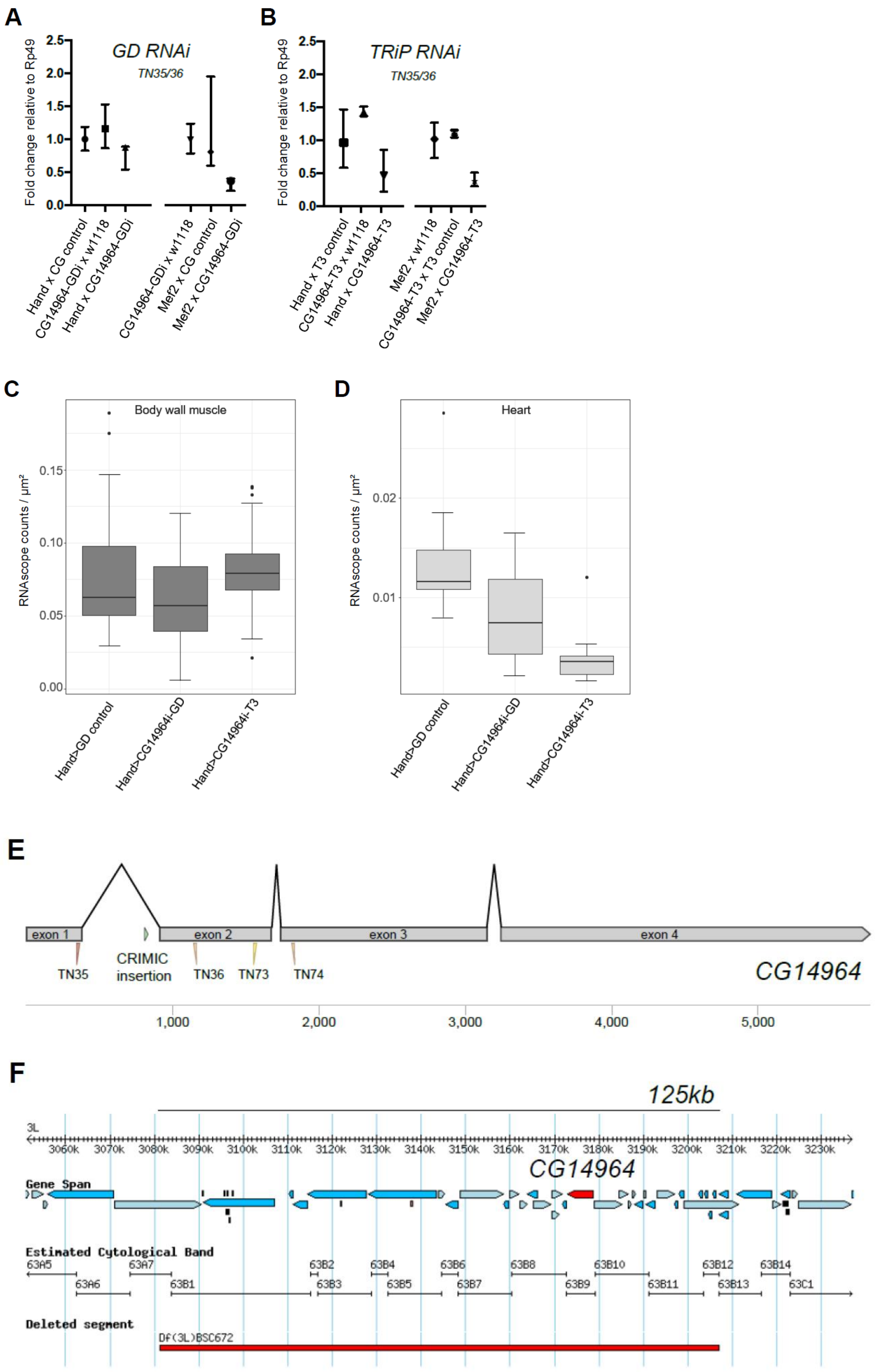

Disease Models & Mechanisms • Supplementary information

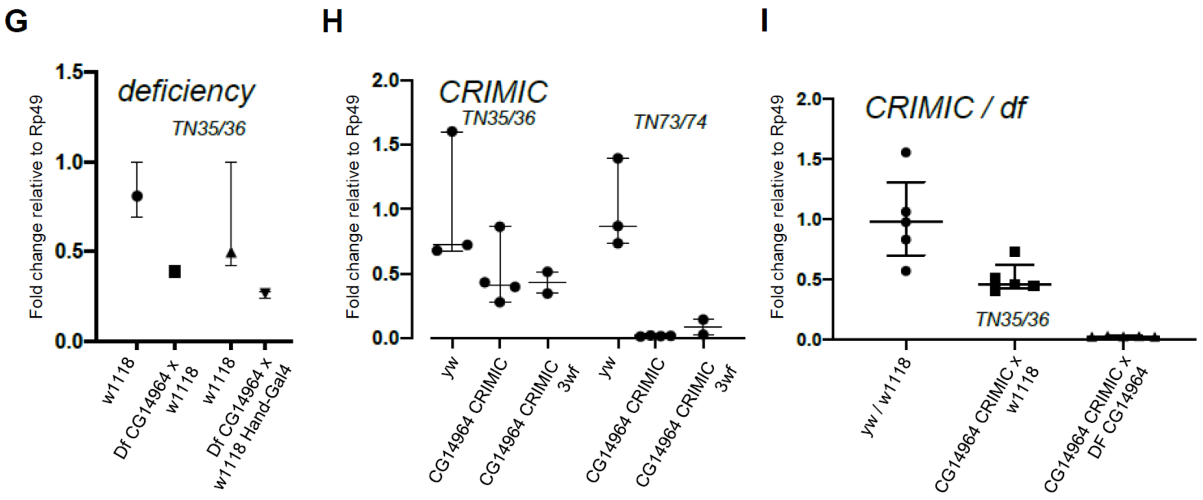

Fig. S6: *CG14964* genomic locus and knockdown efficiency in different fly mutants. (A-B) RT-qPCR results performed in a heart-specific manner showing a knockdown of *CG14964* in (A) *CG14964i*-GD and (B) *CG14964i*-T3 3 weeks old adult hearts where the knockdown is expressed in heart only (*Hand*$^{4.2}$-Gal4) or muscle and heart (*Mef2*-Gal4). (C-D) Quantification of the amount of *CG14964* transcripts counted in body wall muscles (C) or heart (D) of fixed abdominal samples from *Hand*$^{4.2}$>*CG14964i*-GD and *Hand*$^{4.2}$>*CG14964i*-T3 adult flies normalized to control flies, using RNAscope (ACDbio). (E) Organization of the genomic locus of *CG14964* including CRIMIC insertion site and localization of qPCR primers used. (F) Genomic region covered by Df(3L)BSC672 deficiency spanning multiple genes including *CG14964* (red). (G-I) RT-qPCR results performed from RNA isolated from whole (G) heterozygous deficiency flies, (H) homozygous CRIMIC insertion flies and (I) CRIMIC/Df transheterozygotes.

Disease Models & Mechanisms • Supplementary information

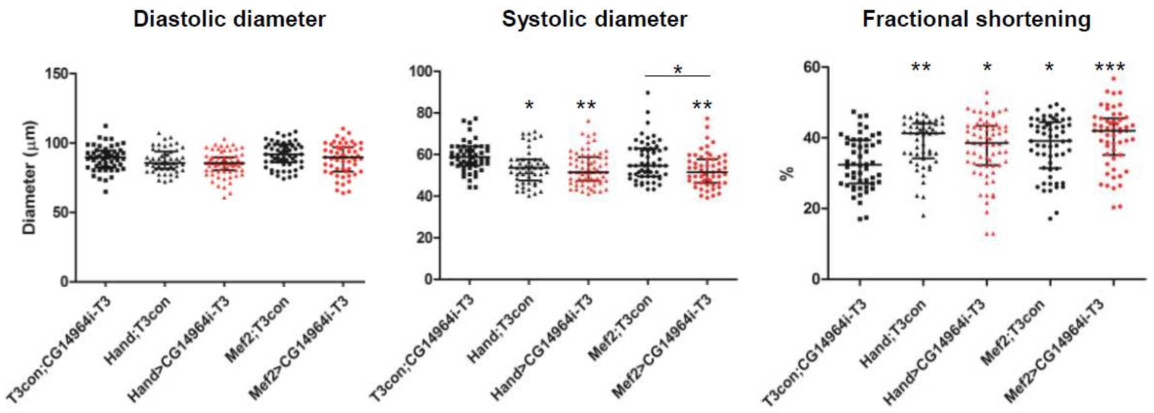

Fig. S7: Cardiac-specific knockdown of *CG14964* leads to heart defects in the adult fly. Cardiac functional analysis on 3 weeks old flies harboring a knockdown of *CG14964* by using CG14964i-T3 shows a trend towards constricted hearts. Shown is the median with interquartile range. Statistics based on one-way ANOVA, Tukey post-hoc test with *p<0.05, ***p<0.01 and ***p<0.001.

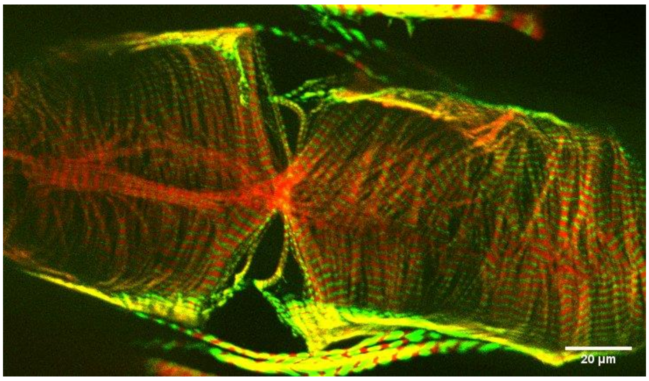

Fig. S8: Representative image showing intact sarcomere structure of 1-week old homozygous *CG14964* CRIMIC mutant (potential null mutant) stained for F-Actin (red) and Mhc (green).

Disease Models & Mechanisms • Supplementary information

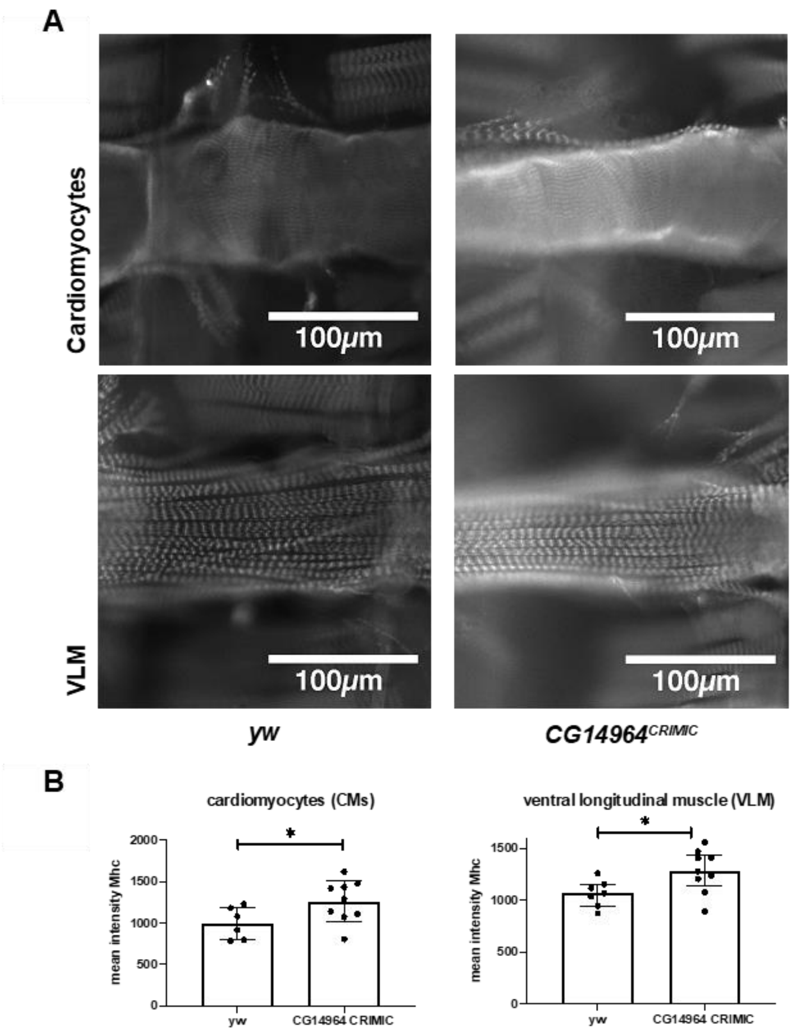

Fig. S9: CG14964 CRIMIC mutants have higher Mhc protein levels compared to controls. Mhc staining of 1-week female *CG14964*^{CRIMIC} flies and controls were stained for Mhc and imaged using identical settings. (A) Mutant hearts have a stronger Mhc signal compared to controls in both, cardiomyocytes and ventral layer muscles (VLM). (B) Mean gray value (intensity) of 5 ROIs per cell type and fly were measured using ImageJ. Graph displays average of mean Mhc intensities per fly Statistics: unpaired t-test. *p≤0.05, **p≤0.005. *CG14964*^{CRIMIC} indicates homozygous *CG14964 CRIMIC* mutants; all intensity values are artificial units.

Disease Models & Mechanisms • Supplementary information

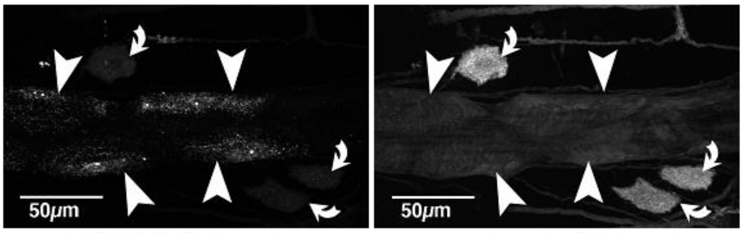

Fig. S10: Overexpression of human MYOM2 using Mef2-Gal4 driver. Tissues are stained for hMYOM2 and pericardin probes. Cardiomyocytes show a clear signal of hMYOM2 (arrowheads), whereas pericardin mRNA is detected in pericardial cells (curved arrows).

Disease Models & Mechanisms • Supplementary information

Patient ID	Sex	Diagnosis	Age at onset (years)	Age at diagnosis (years)	NYHA functional class	Negative T waves (ECG)	Pathological Q waves (ECG)
HCM-01	F	HOCM	31	50	II	Yes	Yes
HCM-02	M	HOCM	19	52	III	Yes	Yes
HCM-03	M	HOCM	50	56	II	Yes	No
HCM-04	F	HOCM	60	69	III	Yes	No

Patient ID	Heart blocks (ECG)	LVEDP (mmHg)	LVEDD (mm)	IVS (mm)	PWT (mm)	SAM (Echo)	Treatment or intervention
HCM-01	No	20	43	16	12	Yes	Medication
HCM-02	No	32	52	15	14	Yes	Myectomy
HCM-03	No	17	50	17	14	Yes	Medication
HCM-04	No	17	48	23	16	Yes	Septal ablation

Table S1. Clinical data of the *MYOM2* mutation carriers with hypertrophic cardiomyopathy (HCM). ECG: Electrocardiogram; M: Male; F: Female; NYHA: New York Heart Association; LVEDP: Left ventricular end-diastolic pressure; LVEDD: Left ventricular end-diastolic diameter; IVS: Interventricular septal thickness; PW: posterior wall thickness; SAM: Systolic anterior movement; HOCM: Hypertrophic obstructive cardiomyopathy

Disease Models & Mechanisms • Supplementary information

SHB Heart code	Age (years)	sex
3.149	56	m
3.073	41	w
5.138	23	m
5.140	31	w
6.008	40	m

Table S2. Details about the samples from the interventricular septum of donor hearts. SHB: Sydney Heart Bank.

Disease Models & Mechanisms • Supplementary information
